# Untargeted Plasma Metabolomic Profiling in Patients with Depressive Disorders: A Preliminary Study

**DOI:** 10.3390/metabo14020110

**Published:** 2024-02-06

**Authors:** Alexander A. Chernonosov, Irina A. Mednova, Lyudmila A. Levchuk, Ekaterina O. Mazurenko, Olga V. Roschina, German G. Simutkin, Nikolay A. Bokhan, Vladimir V. Koval, Svetlana A. Ivanova

**Affiliations:** 1 Institute of Chemical Biology and Fundamental Medicine, Siberian Branch of Russian Academy of Sciences, Lavrentyev Avenue 8, Novosibirsk 630090, Russia; 2 Mental Health Research Institute, Tomsk National Research Medical Center, Russian Academy of Sciences, Aleutskaya Str. 4, Tomsk 634014, Russia; irinka145@yandex.ru (I.A.M.); rla2003@list.ru (L.A.L.); roshchinaov@yandex.ru (O.V.R.); ggsimutkin@gmail.com (G.G.S.); bna909@gmail.com (N.A.B.); ivanovaniipz@gmail.com (S.A.I.); 3 Department of Psychiatry, Addictology and Psychotherapy, Siberian State Medical University, Moskovsky Trakt 2, Tomsk 634050, Russia

**Keywords:** biomarker, depression, metabolomics, metabolome

## Abstract

Depressive disorder is a multifactorial disease that is based on dysfunctions in mental and biological processes. The search for biomarkers can improve its diagnosis, personalize therapy, and lead to a deep understanding of the biochemical processes underlying depression. The purpose of this work was a metabolomic analysis of blood serum to classify patients with depressive disorders and healthy individuals using Compound Discoverer software. Using high-resolution mass spectrometry, blood plasma samples from 60 people were analyzed, of which 30 were included in a comparison group (healthy donors), and 30 were patients with a depressive episode (F32.11) and recurrent depressive disorder (F33.11). Differences between patient and control groups were identified using the built-in utilities in Compound Discoverer software. Compounds were identified by their accurate mass and fragment patterns using the mzCloud database and tentatively identified by their exact mass using the ChemSpider search engine and the KEGG, ChEBI, FDA UNII-NLM, Human Metabolome and LipidMAPS databases. We identified 18 metabolites that could divide patients with depressive disorders from healthy donors. Of these, only two compounds were tentatively identified using the mzCloud database (betaine and piperine) based on their fragmentation spectra. For three compounds ((4S,5S,8S,10R)-4,5,8-trihydroxy-10-methyl-3,4,5,8,9,10-hexahydro-2H-oxecin-2-one, (2E,4E)-N-(2-hydroxy-2-methylpropyl)-2,4-tetradecadienamide and 17α-methyl-androstan-3-hydroxyimine-17β-ol), matches were found in the mzCloud database but with low score, which could not serve as reliable evidence of their structure. Another 13 compounds were identified by their exact mass in the ChemSpider database, 9 (g-butyrobetaine, 6-diazonio-5-oxo-L-norleucine, 11-aminoundecanoic acid, methyl N-acetyl-2-diazonionorleucinate, glycyl-glycyl-argininal, dilaurylmethylamine, 12-ketodeoxycholic acid, dicetylamine, 1-linoleoyl-2-hydroxy-sn-glycero-3-PC) had only molecular formulas proposed, and 4 were unidentified. Thus, the use of Compound Discoverer software alone was not sufficient to identify all revealed metabolites. Nevertheless, the combination of the found metabolites made it possible to divide patients with depressive disorders from healthy donors.

## 1. Introduction

Depression disorders are a group of mental illnesses that seriously impair the social functioning and quality of life of patients and are characterized by a high recurrence rate [1]. The lifetime prevalence of depression in communities from different countries between 1994 and 2014 was 10.8% [2]. Due to the COVID-19 pandemic, the global estimated prevalence of depression in the mid-2020s was seven times higher than in 2017, at 25% [3]. Depression is more common in women, and socioeconomic and family factors have a pronounced influence on its development in both sexes [4]. Despite a large amount of research, the underlying molecular mechanisms of depression remain unclear. Depression is characterized by multifactoriality, heterogeneity of clinical manifestations and lack of diagnostic biomarkers. The classic etiopathological hypothesis is the monoaminergic hypothesis of depression, according to which the neurotransmitters norepinephrine and serotonin are involved in the development of the disease. However, this hypothesis cannot explain the multifactoriality and heterogeneity of the clinical manifestations of depression [5]. Treatment strategies using antidepressants are aimed at normalizing neurotransmitter levels, but some patients with depressive disorders do not achieve an adequate therapeutic response [6]. The existing time lag between treatment administration and therapeutic effect, which can significantly influence the development of the disease, including increasing the likelihood of suicide, also entails the need for additional study of the biological mechanisms of depressive disorders. The cytokine hypothesis (immune-inflammation) of depression has also been intensively studied. According to it, internal or external stress causes an imbalance in the cytokine spectrum, which plays an important role in the severity and continuity of depressive symptoms in vulnerable individuals [7]. The hypothalamic–pituitary–adrenal (HPA) axis hypothesis of depression is also widely discussed. Evidence includes chronically elevated cortisol levels, hypersecretion of corticotropin-releasing factor from the paraventricular nucleus of the hypothalamus, disruption of the HPA axis negative feedback, and adrenal enlargement in depression [8,9]. The inotropic glutamate receptors NMDAR and AMPAR are also closely associated with depression symptoms.

Other theories on the pathophysiology of depression discuss changes in neuroplasticity and neurotrophins in selected vulnerable brain regions; abnormal glutamate and NMDA receptors; a decrease in synaptic plasticity; increased apoptosis; insufficiency of neurosteroid synthesis; disruption of the endogenous opiate system; and changes in mitochondrial function and neuronal bioenergetics [10,11,12,13,14].

The identification of potential biomarkers of mental illness is of particular interest in metabolomic research due to the complex and multifaceted changes in biochemical pathways that lead to the pathological state. Metabolomics has become the basis for the system-wide profiling of diseases and medicine because of the interest in and the recognition of the important role of metabolites in biological processes and the dynamic influence of the metabolome on biological systems. Between 2007 and 2017, the number of publications mentioning the terms ‘metabolomic’ and ‘biomarker’ increased significantly [15], and this increase has continued in recent years, with the number of publications doubling since 2017. This growth has been attributed to advances in liquid chromatography–mass spectrometry (LC-MS), which has allowed the untargeted metabolomic analysis of biological fluids [15,16,17]. Several types of mass spectrometers, such as time-of-flight (TOF), ion trap TOF, hybrid quadrupole TOF, and Orbitraps, routinely achieve high mass accuracy [15,18,19]. This enables the determination of a compound’s molecular formula from its exact mass. However, due to their absence in reference databases, the wide range of metabolite concentrations, and the limitations of mass spectrometry data acquisition speed, compound identification remains poor, and many peaks remain unidentified [15].

A variety of free software is available for compound annotation, both as an online resource and implemented using the R and Python programming languages [20,21,22,23]. Moreover, each mass spectrometer company provides its own software to process, annotate and visualize mass spectrometry data, for example, Progenesis QI (Waters, Milford, MA, USA), XCMSplus (Sciex, Framingham, MA, USA), MassHunter (Agilent, Palo Alto, CA, USA), and Compound Discoverer (Thermo Scientific, San Jose, CA, USA). The last one works only on .raw files generated by Xcalibur software and Orbitrap mass spectrometers but provides automated compound annotation via the mzCloud database [24]. Compound Discoverer was used in various untargeted metabolomics studies for, e.g., compound identification in ethanol extract of plants [25,26,27,28], comparing samples of propolis [29] and lentil seed coats [30], determining the metabolic changes during the browning process [31], the analysis of biological fluid samples [32,33], autism studies, [34] etc.

The search for biomarkers can improve disease diagnosis, personalize therapy and lead to a deep understanding of the biochemical processes underlying depression. Metabolomic approaches based on the use of gas chromatography–mass spectrometry and LC-MS are widely used to search for potential biomarkers of depression [35]. According to systematic analyses in these studies, changes in metabolites have been observed in depressive disorders and play a role in amino acid metabolism, energy and lipid metabolism and cell signaling, as well as affect cell membrane components, neurotransmitters, inflammatory and immunological mediators, hormone activators and precursors, and sleep regulators [35,36,37]. A systematic review summarized data indicating that in patients with depression, the levels of lipids (arachidonic, linoleic, oleic, heptadecylic and valeric acids and cholesterol), phosphoethanolamine, histamine, leucine-enkephalin, amino acids (tryptophan, kynurenine, gamma-glutamylleucine, arginine and isoleucine) and inosine in plasma/serum are reduced. Increased concentrations of gamma-aminobutyric acid, dopamine, xanthine, adenosine α-1-acid glycoprotein 1 and leucine-rich α-2-glycoprotein have also been found in the plasma/serum of patients with depressive disorders [36]. At the same time, researchers noted some difficulties associated with the ambiguous reproducibility of results, and therefore new studies on metabolomic profiling remain relevant [35].

Therefore, the aims of the work were to classify patients with depressive disorders and healthy individuals using metabolomic analysis of blood serum and to evaluate whether this could be achieved with Compound Discoverer alone.

## 2. Materials and Methods

### 2.1. Chemicals and Reagents

HPLC-grade methanol and LC-MS-grade acetonitrile were purchased from J. T. Baker (Gliwice, Poland) and Biosolve (Dieuze, France), respectively. Formic acid was acquired from Sigma-Aldrich (St. Louis, MO, USA). HPLC-grade water was produced with a Milli-Q purification system from Millipore Corp. (Bedford, MA, USA).

### 2.2. Study Population and Sample Collection

The study was conducted in accordance with the Declaration of Helsinki of the World Medical Association. The study protocol was approved by the Ethics Committee of the Mental Health Research Institute, Tomsk National Research Medical Center, the Russian Academy of Sciences (approval on 17 June 2022, #154). Clinical examination was carried out in accordance with the Mini-International Neuropsychiatric Interview (M.I.N.I.) for DSM-5, Russian version. Thirty patients with depressive disorders were included in the study after signing an informed consent. There were 14 patients with a depressive episode and 16 participants with recurrent depressive disorders according to the International Statistical Classification of Diseases and Related Health Problems, 10th Revision (ICD-10: F32.11 and F33.11). The patients were recruited from the Affective States Department in the Mental Health Research Institute of the Tomsk National Research Medical Center. The control group consisted of healthy volunteers (*n* = 30). The selection of healthy volunteers was carried out among the staff and students at the Mental Health Research Institute and Siberian State Medical University. All control group participants were assessed by a trained psychiatrist to determine whether they met the inclusion/exclusion criteria and by a self-report questionnaire. The questionnaire screened for both physical and mental pathologies, e.g., endocrine, neurological, gynecological and psychiatric disorders.

The inclusion criteria for healthy volunteers were age from 18 to 55 years, consent to participate in the study, absence of mental disorders, and absence of somatic disorders requiring intensive treatment. The inclusion criteria for patients were a diagnosis of depressive disorders (F32.11, F33.11) according to ICD-10 and age of 18–55 years. We excluded patients with other comorbid mental disorders, for instance, schizophrenia, intellectual disability, alcoholic psychoses, and patients with acute physical diseases. The screening for relevant pathologies for the in-/exclusion of subjects, disease development and the severity of the conditions was performed according to clinical assessment by three trained psychiatrists on the first day of admission.

The basic demographic and clinical data are presented in Table 1.

### 2.3. Sample Preparation

Blood from a peripheral venous was collected in Vacutainer tubes with a clotting activator from each study participant between 8 and 9 a.m., after 12 h of overnight fasting and before the intake of any food or medication. Then, the blood was centrifuged at 2000 rcf for 20 min at 4 °C. A 50 μL aliquot of each serum sample was placed in a 1.5 mL Eppendorf tube. Then, 50 μL of methanol was added, and the samples were vortexed approximately for 1 min to obtain a fine suspension of serum proteins. Next, 150 μL of acetonitrile was added, and the samples were shaken for 10 min at 25 °C and 900 rpm. After centrifugation at 13,000× *g* for 10 min, 100 μL of the supernatants was transferred into glass vials for subsequent injection into an ultra-high-performance liquid chromatography (UPLC) system.

### 2.4. LC/MS Parameters

The mass spectrometric analysis was carried out at the Core Facility of Mass Spectrometric Analysis at the Institute of Chemical Biology and Fundamental Medicine, the Siberian Branch of the Russian Academy of Sciences. The prepared samples were analyzed by a DIONEX UltiMate 3000 HPLC system and a Q-Exactive HF high-resolution tandem mass spectrometer (Thermo Fisher Scientific, Inc., Waltham, MA, USA). A ProntoSil-120-3-C18 column (2 mm × 75 mm, 3 μm, EcoNova, Novosibirsk, Russia) with an Eclipse XBD-C18 guard column (4.6 mm × 12.5 mm, 5 μm) was used for reversed-phase separation. The temperature of the column was maintained at 25 °C. The mobile phase consisted of water containing 0.1% FA (A) and acetonitrile containing 0.1% FA (B); the flow rate was 0.3 mL/min. The chromatographic separation of all substances was performed in a 15 min run using the following gradient elution program for the mobile phase: 80% buffer B from minute 0 to minute 10, 1% buffer B to minute 12, 80% buffer B from minute 12.1 to minute 15.

The Q-Exactive HF orbitrap mass spectrometer (Thermo Fisher Scientific, Inc.) was utilized to analyze the metabolites in positive ion mode. The settings of the ESI source were as follows: 4.2 kV electrospray voltage; capillary temperature, 320 °C; and S lens RF level, 50. Precursor spectra (*m*/*z* 100–1035) were collected at 45,000 resolution at *m*/*z* 200 to hit an automatic gain control (AGC) target of 1 × 10^5^ with a maximum injection time of 100 ms. A top 5 configuration to acquire data with isolation windows *m*/*z* 1.4 was set in data-dependent acquisition (DDA) mode. The resolution was set at 15,000 to collect fragment spectra for achieving an AGC target of 1 × 10^5^, and the maximum injection time was set to 65 ms. The normalized collision energies of 20, 40, and 80 eV were set for whole molecule fragmentation. The data were acquired using Xcalibur 4.0 software (Thermo Fisher Scientific). All samples were analyzed in three technical replicates.

### 2.5. Analysis Optimization

When conducting comparative metabolomic analysis, it is crucial to adhere to the same conditions for sample preparation and data collection. Additionally, it is necessary to examine the stability of the samples after extraction and before data collection. Given the large number of compounds with different chemical characteristics, it is possible that some compounds will be degraded. In this case, differences between samples will be primarily caused by heterogeneity in the analysis conditions rather than by different concentrations of compounds in the groups.

To study stability, we analyzed the same test sample every hour and studied the change in signal area for the identified compounds in Compound Discoverer. The results showed that after extraction, the samples remained stable for 6 h.

The chromatographic separation parameters were determined in the next step. An isocratic gradient for 12 min was found to reveal the highest number of compounds in the subsequent analysis using the Compound Discoverer program, while maintaining a minimum chromatography time. Therefore, the samples were divided into groups of six, each consisting of three patient samples and three healthy donor samples. Each sample was analyzed three times to ensure accuracy, and including the washes, the total analysis time did not exceed six hours.

### 2.6. Compound Identification and Statistical Analysis

Following their acquisition, the mass spectrometry data (.raw files) were inputted into Compound Discoverer 3.3 software. In the “Study Definition”, the samples were divided by the categorical factors “Diagnosis” and “Sample name”, and technical replicates were specified as biological replicate factor. The following ratios were generated at the “Grouping and Ratios” step: patients with depressive episode vs. control group (F3211/healthy), patients with depressive episode vs. control group (F3311/healthy), and patients with depressive episode vs. patients with depressive episode (F3311/F3211). A common workflow “Untargeted Metabolomics with Statistics Detect Unknowns with ID using Online Databases” was used for compounds identification, with mass tolerance of 10 ppm in all appropriate nodes. Several databases, i.e., ChEBI (https://www.ebi.ac.uk/chebi/; accessed on 5 December 2023), FDA UNII–NLM (https://precision.fda.gov/uniisearch/; accessed on 5 December 2023), Human Metabolome Database (https://hmdb.ca/; accessed on 5 December 2023), KEGG (https://www.genome.jp/kegg/; accessed on 5 December 2023), and LipidMAPS (https://www.lipidmaps.org/; accessed on 5 December 2023), were chosen in the ChemSpider search node.

Several mzVaults were used in the mzVault search node, i.e., Negative ion mode_Jan2021.db and Positive ion mode_Jan2021.db (https://more.bham.ac.uk/bamcg/resources/; accessed on 5 December 2023), MS2_library.db [38], LipidBlast-V56B-Neg.db, and LipidBlast-V56B-Pos.db [39]. The metabolites were identified on the basis of both accurate mass and fragment mass “fingerprint” spectra via searches against the spectra of compounds available in the mzCloud database (https://www.mzcloud.org; accessed on 5 December 2023) and local mzVaults databases. Compounds that were absent in mzCloud were tentatively identified using a ChemSpider search.

Statistical analysis was carried out using modules built in Compound Discoverer 3.3, i.e., principal component analysis and differential analysis. The *p*-value per group ratio was calculated by a multivariate paired *t*-test (assuming equal variance). Background signals with a mean area less than 3 × 10^6^ were filtered out, resulting in a total of 1573 compound lines (some of the compounds were presented in multiple lines). The PCA tool built into Compound Discoverer was used to separate groups, but the initial set of compounds did not result in meaningful group separation. The built-in differential analysis utility was used to identify compounds to separate the patient groups from the control group. We selected compounds whose concentrations were significantly different (*p*-value < 0.05) by more than 2-fold in the patients and control groups. Then, we filtered the results by *p*-value adjusted using Benjamini–Hochberg correction for the false discovery rate, with adj. *p*-value < 0.05 for at least one patient group. And additional manual filtering was performed.

## 3. Results and Discussion

**Figure 1:**
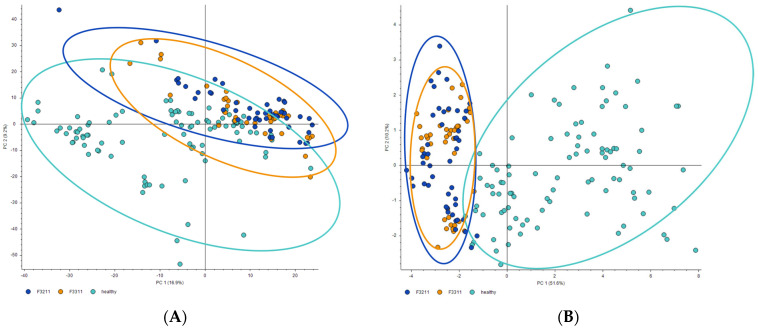
PCA score plots of the MS data before (**A**) and after filtering (**B**), constructed in the Compound Discoverer software using Pareto scaling for patients with depressive disorders (F3211, blue points) and recurrent depressive disorders (F3311, orange points) and the control group (healthy, cyan points).

Although about 1500 compounds were identified by the Compound Discoverer analysis, the multivariable analysis of the full set of compounds failed to fully discriminate between patient groups and healthy donors. Moreover, the first two principal components (PCs) demonstrated in the PCA score plot slight variations, with PC1 and PC2 being 16.9% and 9.2%, respectively (Figure 1A). To differentiate between patients and controls, 18 compounds were selected as a result of sequential filtration (Table 2). In the PCA score plot, the variation of the first component PC1 increased significantly to 51.6%, even though the variation of the second component PC2 hardly increased and reached 10.2% (Figure 1B). Only 4 from the selected 18 compounds (**1**, **6**, **8**, **10**) were tentatively identified in the mzCloud database from their fragment patterns, and for compound **9**, fragmentation similar to that of a different molecule was observed. Two compounds were identified with good mzCloud score (Table 2): compound **1** as betaine, and compound **8** as piperine. The comparison of the experimental fragmentation spectra with the spectra from the mzCloud database is presented in Figure S1 for betaine and in Figure S2 for piperine.

Because of poor fragmentation for compounds **6** (Figure S3) and **10** (Figure S4), the suggested structures were most likely incorrect. This was also evidenced by the low mzCloud scores of 60.2 for compound **6** and 55.2 for compound **10** (Table 2). The fragmentation pattern of compound **9** was similar to that in the autoprocessed spectrum of NP-008993, with different molecular weight of 314.24571 and gross formula C_18_H_34_O_4_ (Figure S5). Nevertheless, such fragmentation could correspond to decadienamide or sphingosine derivatives. Other compounds were not found in the mzCloud database and were tentatively identified only by their accurate mass using a ChemSpider search, suggesting their possible association with neurodegenerative diseases. Compounds **13** and **14** had different retention times but the same molecular weight.

According to a meta-analysis, animal models of depression showed decreased levels of neurotransmitters and increased levels of kynurenine in the brain; a decrease in the concentration of amino acids and an increase in the level of corticosterone in the blood; and signs of unbalanced energy metabolism and microbial metabolites in urine [5]. This correlated with the decreased concentrations of compounds **3**, **5** and **7**, tentatively identified as norleucine derivatives and three-amino acid peptide, respectively, in patients with depressive disorders. A large-scale meta-analysis including 10,145 control subjects and 5283 persons with depression indicated a distinctive profile of circulating lipid metabolites associated with depression [37]. Compounds **13** and **14**, identified as dicetylamine, could be part of a lipid molecule with an amino group and an aliphatic chain. Also compounds **15** and **17**–**19**, which presented identical fragments, could be part of an iterative molecule.

Betaine (compound **1**) takes part in the conversion of homocysteine to methionine. Disruption of these metabolic pathways leads to the accumulation of homocysteine. Excess homocysteine, hyperhomocysteinemia and homocystinuria are associated with various somatic and mental disorders: arterial occlusive disease, hypertriglycerilemia, venous thrombosis, chronic renal failure, megaloblastic anemia, osteoporosis, Alzheimer’s disease and cognitive decline [40,41,42,43]. According to a systematic review and meta-analysis, elevated homocysteine levels are positively associated with the risk of depression [44]. The homocysteine-converting role of betaine is hypothesized to be critical for homeostasis [45]; when betaine levels decrease, which is accompanied by an increase in homocysteine levels and a decrease in methionine production, the synthesis of dopamine, serotonin and norepinephrine decreases, which leads to depression. In addition, homocysteine has a direct toxic effect on endothelial cells and cerebral vascular neurons [46,47]. In a metabolomics analysis of plasma using liquid chromatography–mass spectrometry, betaine was one of five metabolites that were associated with depression severity in three independent cohorts, independent of the presence or absence of medical treatments and diagnostic differences [48]. The plasma betaine levels were also reduced in patients with post-stroke depression [46]. A number of studies demonstrated that a combination therapy consisting of betaine and S-adenosylmethionine was effective and safe in patients with mild to moderate depression [49,50].

In recent years, research studying the brain–gut microbiological axis in mental disorders have become widespread. In particular, significant differences in the intestinal microbiota have been identified in patients with depression compared to healthy people [51,52,53,54,55]. Cholic acid and its derivatives (compound **12**) are bile acids that are synthesized in the liver and then processed by intestinal bacterial enzymes. Bile acids can change the permeability of the BBB and influence inflammation processes and oxido-nitrosative and endoplasmic reticulum stress [56,57]. Significant alterations in the content of cholic acid derivatives were revealed in patients with depression compared to healthy people. [56,58]. Using targeted metabolomics, it was shown that changes in the bile acid profile in patients with major depressive disorder were associated with higher levels of anxiety and an increased likelihood of first-line treatment failure [59].

The decrease in g-butyrobetaine (trimethylammoniobutanoic acid, compound **2**) levels in depressed patients can be explained in several ways. g-Butyrobetaine is a trimethylated derivative of y-aminobutyric acid (GABA) [60]. GABA is the main inhibitory neurotransmitter of the central nervous system; decreased GABA levels are associated with depression and mood disorders [61,62]. Animal studies suggest that the gut microbiota may alter GABA activity in the brain via the vagus nerve in animals. On the other hand, g-butyrobetaine is a precursor to L-carnitine in mammal endogenous synthesis; in humans, endogenous synthesis accounts for 25% of L-carnitine, while 75% of L-carnitine derives from the diet [63]. L-carnitine is a major participant in energy metabolism involved in the β-oxidation of fatty acids [64]. There was a decrease in the level of L-carnitine in the plasma of patients with depression compared to healthy individuals [65,66]. In addition, L-carnitine and acetyl-L-carnitine were shown to be markers of treatment effectiveness in depression [66].

1-Linoleoyl-2-hydroxy-sn-glycero-3-PC (LGPC, compound **16**) is a lysophospholipid containing linoleic acid. Insulin resistance and dysglycemia lead to decreased LGPC levels in human serum [67]. In addition, we found unidentifiable compounds, most likely representing fatty acid residues. Given recent meta-analyses showing significant relationships between metabolic disorders, insulin resistance and depression [68,69], it can be assumed that the appearance of these compounds in the metabolomic signature confirmed the close relationship between these mental and somatic conditions.

Compound **8**, identified as piperine by multiple fragments with a high mzCloud score (87.5), is not an endogenous metabolite. Piperine is one of the main constituents of pepper, natural substances of alkaloid origin with multiple pharmacological properties. These include anti-inflammatory [70], antimicrobial, antioxidant [71], hepatoprotective [72], anticonvulsant [73], and neuroprotective [74] activities. Piperine also showed potential for treating depressive disorders and enhancing memory in animal models [75,76]. The oral administration of piperine, the main alkaloid of black pepper, was shown to be effective with respect to antidepressant activity and cognitive effects in rat experiments [76]. Its effects were shown to be comparable to those of the positive controls fluoxetine and donepezil hydrochloride. Interestingly, piperine does not cause false positives in the effort swimming test, as it has no stimulatory effect on locomotor activity. Thus, the antidepressant activity of piperine is not a false positive effect. Similar results were obtained in another study, which also demonstrated the antidepressant activity of piperine in rats under chronic mild stress [75]. In several mouse models of behavioral despair, piperine was shown to inhibit monoamine oxidase activity, increase monoamine neurotransmitter levels, and thus produce antidepressant-like activity [77]. The antidepressant effects of piperine were associated with increased proliferation of hippocampal progenitor cells [75]. It was also shown that brain-derived neurotrophic factor signaling mediates this antidepressant-like effect of piperine in chronically stressed mice [78]. Since piperine is ingested with food and affects serotonin production, it is not clear from our study whether a reduction in black pepper intake negatively affects the serotonin levels or whether patients with depressive episodes dislike peppered foods.

The remaining compounds we tentatively identified are not endogenous and are metabolites that reflect the likely influence of environmental factors. We controlled blood sampling in the study participants, trying to ensure equilibrium concentrations of metabolites in the blood. Understanding the role of these compounds for a holistic deciphering of the molecular mechanisms that control the final phenotype seems to be possible to realize in future studies, as knowledge about the human exposome accumulates and expands.

The comparison of our results with data from other untargeted metabolomics studies of depression led to mixed conclusions. In general, a fairly large amount of knowledge has been accumulated about changes in the metabolite profile in patients with depressive disorders [35,36,37]. However, the results of these studies are generally contradictory. According to a recent systematic review, the most consistent evidence was obtained for kynurenine and acylcarnitine [36]. In our study, we did not find changes in these metabolites, but their derivatives were identified as altered in depressive disorders. The lack of clear reproducibility of the results of metabolomic studies is associated both with the high heterogeneity of the clinical manifestations of the disease and with different methodological approaches in different studies. Therefore, work in this direction, conducting similar studies including a detailed description of the methodologies used, can contribute to the development of metabolomics approaches and, in the future, to the acquisition of true knowledge.

The main limitations of our study are the small sample size and the tentative identification of most of the revealed metabolites using only Compound Discoverer software.

## 4. Conclusions

Eighteen compounds were revealed as metabolites with significantly different levels in patients with depressive disorders in comparison with healthy controls through untargeted metabolomics using Compound Discoverer software. The structure of two compounds was confirmed by their fragmentation patterns through the mzCloud database, while the remaining compounds were identified tentatively. Although the utilities built in Compound Discoverer software were sufficient to identify differences in metabolites between groups, Compound Discoverer was not sufficient to reliably identify all detected compounds. It can be used for the initial identification of ions whose amounts differ meaningfully between groups, but reference standards are required for the accurate identification of presumed molecules. Even without knowing the exact structure of the proposed compounds, using multivariate analysis of mass spectrometry data, the described set of metabolites could be useful in a clinical test to reveal patients with depressive disorders. Further research is required to conclusively identify and validate the revealed metabolites as potential biomarkers of depressive disorders.

## Figures and Tables

**Table 1:** Demographics and clinical characteristics of the study population, Me (Q1; Q3).

Indicators	Patients with Depressive Disorders				Healthy Controls	*p*-Value
	Overall	Depressive Episode (F32.11)	Recurrent Depressive Disorders (F33.11)	*p*-Value		
Age, years	40.5 (37; 48)	42 (39; 49)	39 (37; 45,5)	0.759	40 (29; 47)	0.359
Gender (male, n (%)/female, n (%))	2(6.7%)/28(93.3%)	1(7.1%)/13(92.9%)	1(6.7%)/15(93.3%)	0.922	2(6.7%)/28(93.3%)	1.0
Duration of disease, years	0.67 (0.33; 4.5)	0.42 (0.25; 0.58)	5 (3.5; 10)	0.0001 *	-	-
Number of depressive episodes experienced (excluding the current one)	2 (2; 2)	0	2 (1.5; 2.5)	0.0001 *	-	-
Duration of the current affective episode, months	-	6 (3; 10)	3 (2; 8)	0.089	-	-
BMI	25.1 (22.3; 27.3)	25.1 (22.9; 27.4)	25.1 (21.6; 27.1)	0.786	24.7 (22.5; 28.8)	0.531

BMI—body mass index; * *p*-value < 0.05—statistically significant difference between groups. Comparisons between groups were performed using the chi-squared test for gender and the Mann–Whitney U-test for the other indicators.

**Table 2:** Compounds whose levels differed significantly between patients with depressive disorders and healthy volunteers.

No.	Calc. MW, Da	Formula	Name	mzCloud Score	F3211/ Healthy (*p*-Value)	F3311/ Healthy (*p*-Value)
1	117,07860	C_5_H_11_NO_2_	Betaine	93.2	0.37 (0.0003)	0.31 (0.0017)
2	145,10947	C_7_H_15_NO_2_	g-Butyrobetaine	-	0.56 (0.002)	0.53 (0.015)
3	172,07044	C_6_H_10_N_3_O_3_	6-Diazonio-5-oxo-L-norleucine	-	0.39 (0.0006)	0.39 (0.002)
4	201,17209	C_11_H_23_NO_2_	11-Aminoundecanoic acid	-	0.07 (0.002)	0.08 (0.004)
5	214,11747	C_9_H_16_N_3_O_3_	Methyl N-acetyl-2-diazonionorleucinate	-	0.31 (0.004)	0.30 (0.002)
6	216,09639	C_10_H_16_O_5_	(4S,5S,8S,10R)-4,5,8-trihydroxy-10-methyl- 3,4,5,8,9,10-hexahydro-2H-oxecin-2-one	60.2	0.36 (0.001)	0.42 (0.005)
7	272,15857	C_10_H_20_N_6_O_3_	Glycyl-glycyl-argininal	-	0.07 (0.00007)	0.08 (0.001)
8	285,13511	C_17_H_19_NO_3_	Piperine	87.5	0.59(0.03)	0.51 (0.005)
9	295,24900	C_18_H_33_NO_2_	(2E,4E)-N-(2-Hydroxy-2-methylpropyl)- 2,4-tetradecadienamide	52.2 *	7.2 (0.0007)	8.8 (0.003)
10	319,24639	C_20_H_33_NO_2_	17α-Methyl-androstan-3-hydroxyimine-17β-ol	55.2	20.3 (0.0004)	26.2 (0.0002)
11	367,41679	C_25_H_53_N	Dilaurylmethylamine	-	0.28 (0.003)	0.37 (0.003)
12	390,2752	C_24_H_38_O_4_	12-Ketodeoxycholic acid	-	0.42 (0.005)	0.34 (0.007)
13	465,52609	C_32_H_67_N	Dicetylamine	-	0.02 (0.004)	0.02 (0.006)
14	465,52684	C_32_H_67_N	Dicetylamine	-	0.11(0.1)	0.13(0.1)
15	497,89136	C_8_H_7_N_2_O_17_PS_2_	-	-	0.48 (0.002)	0.54 (0.014)
16	519,33092	C_26_H_50_NO_7_P	1-Linoleoyl-2-hydroxy-sn-glycero-3-PC	-	0.17 (0.006)	0.14 (0.0006)
17	701,85267	C_12_H_5_N_10_O_16_P_3_S_2_	-	-	0.49 (0.002)	0.57 (0.019)
18	837,82763	-	-	-	0.46 (0.0005)	0.54 (0.012)
19	905,81407	-	-	-	0.47 (0.002)	0.44 (0.013)

* mzCloud best similarity score.

## Data Availability

Data are available on request, owing to privacy and ethical restrictions.

## Supplementary Materials

The following supporting information can be downloaded at https://www.mdpi.com/article/10.3390/metabo14020110/s1, Figure S1: Comparison of the experimental fragmentation spectrum of betaine (top) with the spectrum from the mzCloud database (bottom); Figure S2: Comparison of the experimental fragmentation spectrum of piperine (top) with the spectrum from the mzCloud database (bottom); Figure S3: Comparison of the experimental fragmentation spectrum of (4S,5S,8S,10R)-4,5,8-trihydroxy-10-methyl-3,4,5,8,9,10-hexahydro-2H-oxecin-2-one (top) with the spectrum from the mzCloud database (bottom); Figure S4: Comparison of the experimental fragmentation spectrum of 17α-Methyl-androstan-3-hydroxyimine-17β-ol (top) with the spectrum from the mzCloud database (bottom); Figure S5: Comparison of the experimental fragmentation spectrum of (2E,4E)-N-(2-Hydroxy-2-methylpropyl)-2,4-tetradecadienamide (top) with the spectrum of NP-008993 from the mzCloud database (bottom).
