# Supplementary material for: Untargeted Plasma Metabolomic Profiling in Patients with Depressive Disorders: A Preliminary Study
Source: Metabolites. 2024 Feb 6;14(2):110. doi: 10.3390/metabo14020110 (PMC10890195; doi:10.3390/metabo14020110)
Supplement: Supplementary file 1 [file metabolites-14-00110-s001.zip › metabolites-2827630-supplementary.pdf]

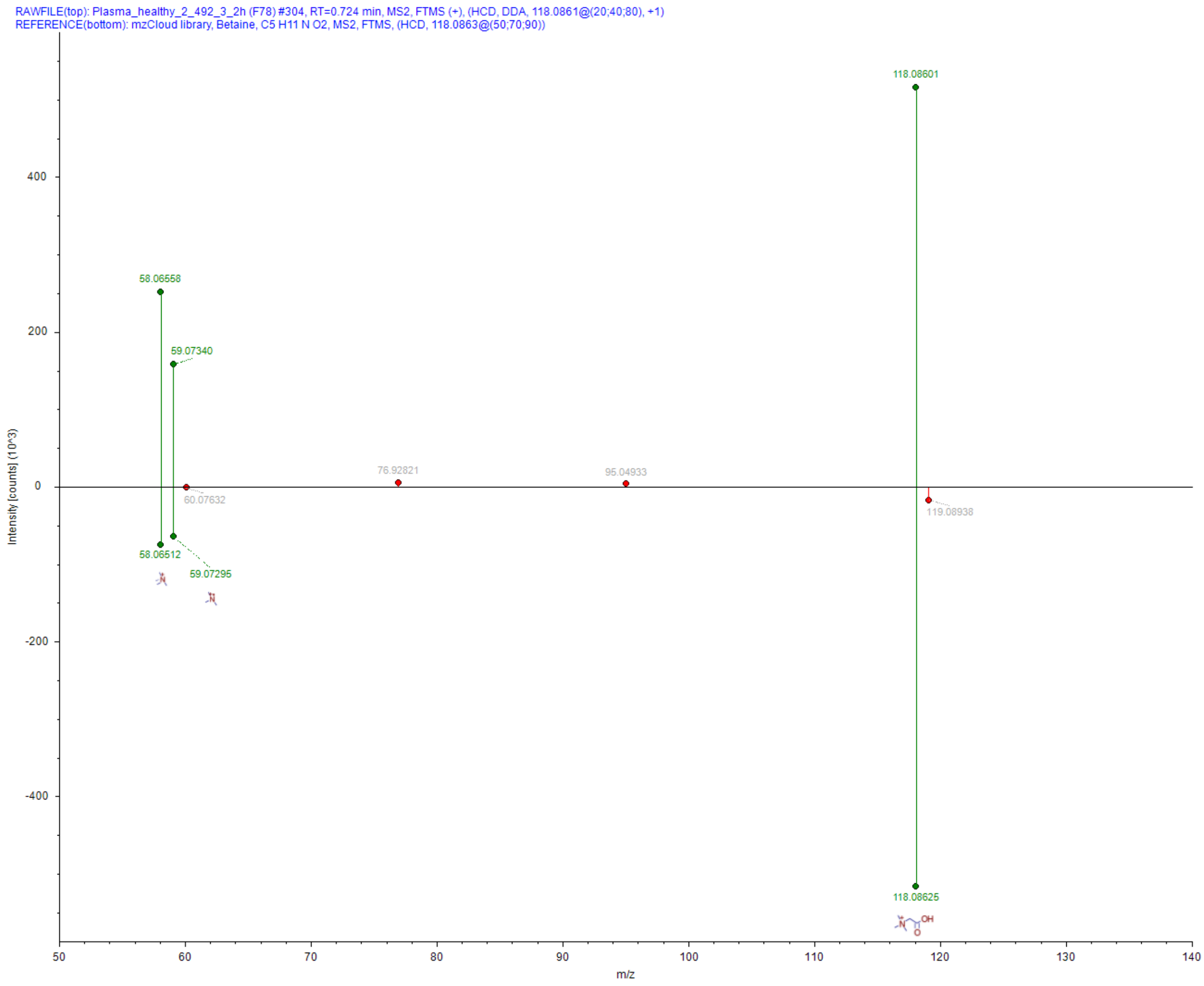

Figure S1. Comparison of the experimental fragmentation spectrum of betaine (top) with the spectrum from the mzCloud database (bottom).

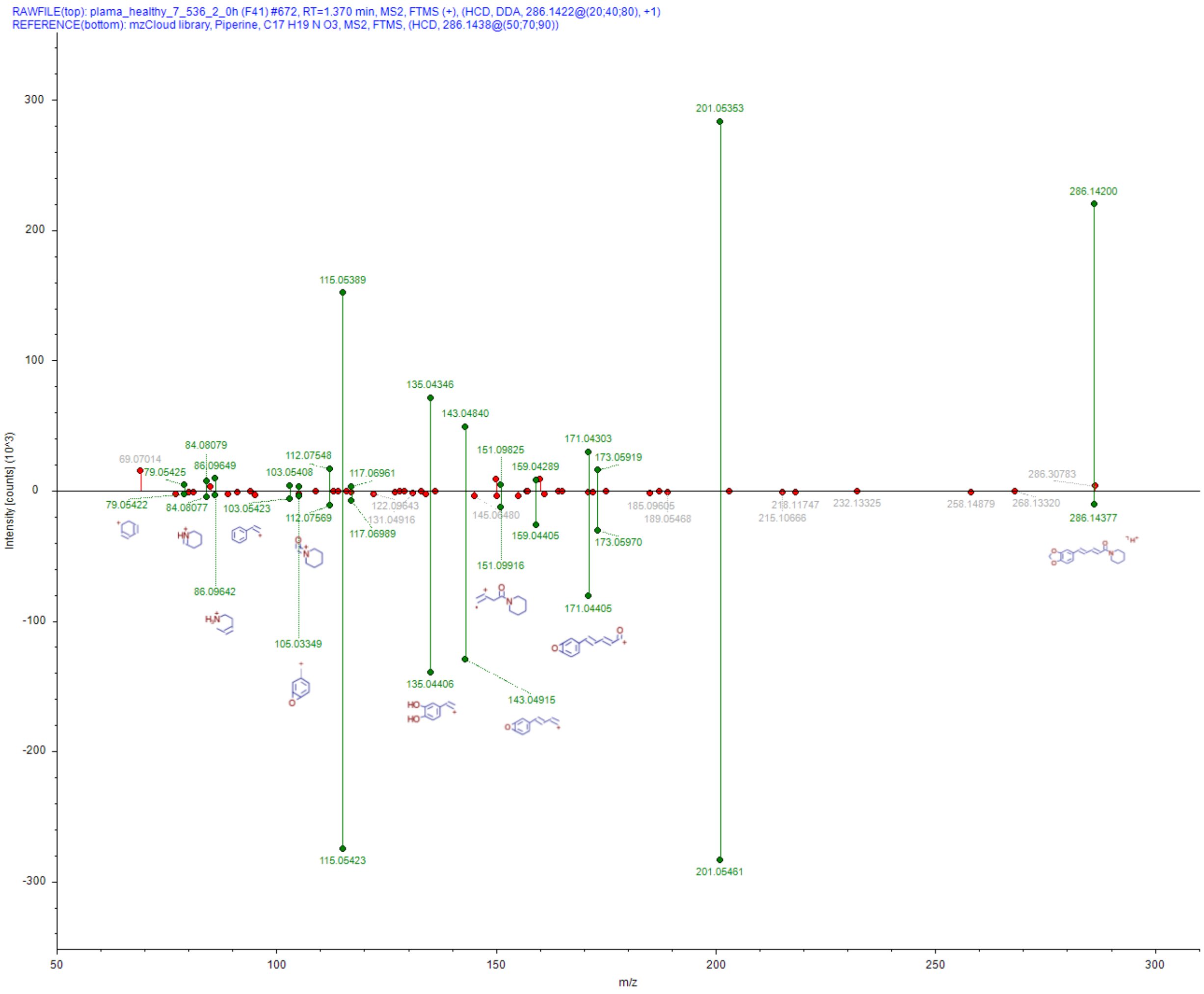

Figure S2. Comparison of the experimental fragmentation spectrum of piperine (top) with the spectrum from the mzCloud database (bottom).

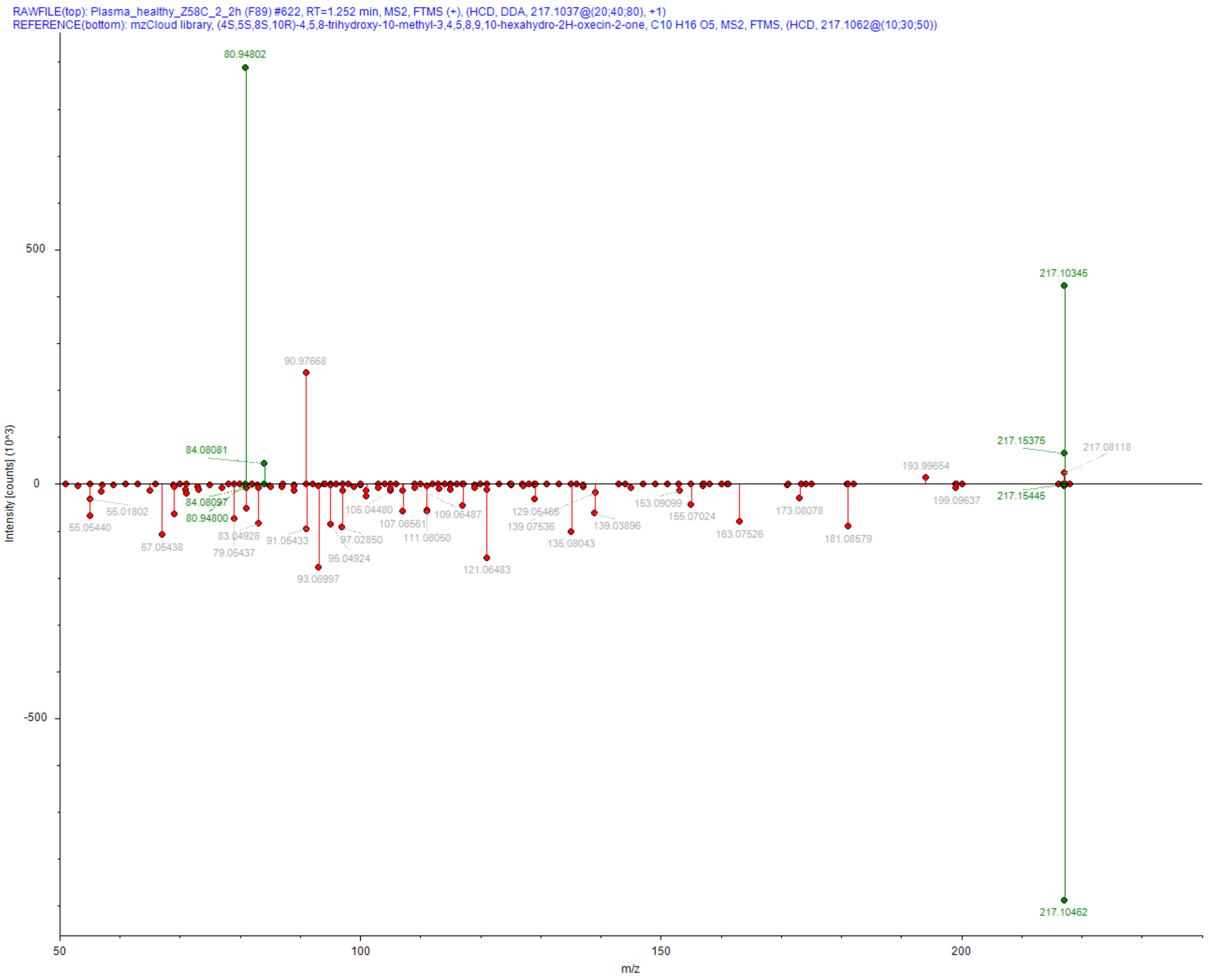

Figure S3. Comparison of the experimental fragmentation spectrum of (4S,5S,8S,10R)-4,5,8-trihydroxy-10-methyl-3,4,5,8,9,10-hexahydro-2H-oxecin-2-one (top) with the spectrum from the mzCloud database (bottom).

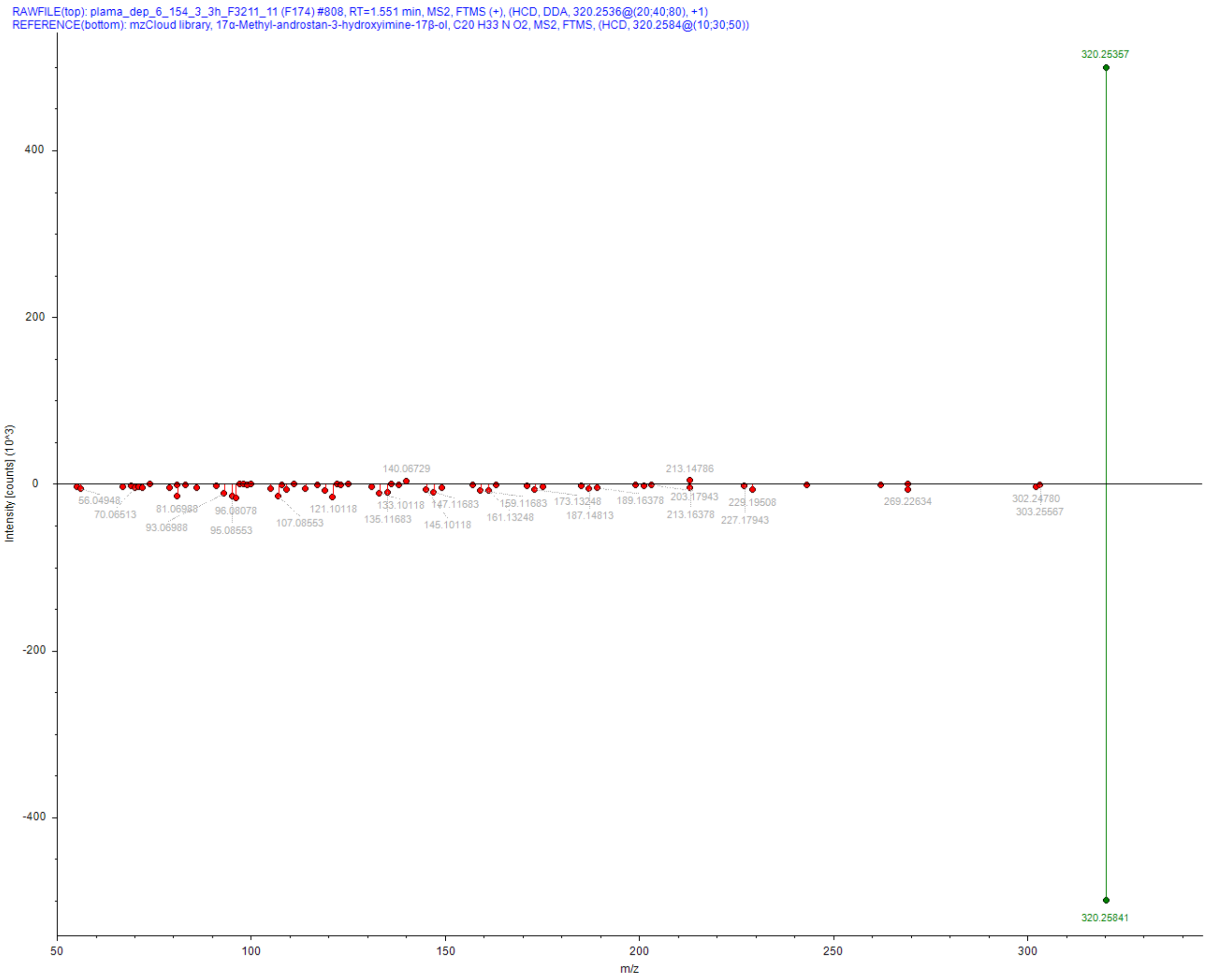

Figure S4. Comparison of the experimental fragmentation spectrum of 17α-Methyl-androstan-3-hydroxyimine-17β-ol (top) with the spectrum from the mzCloud database (bottom).

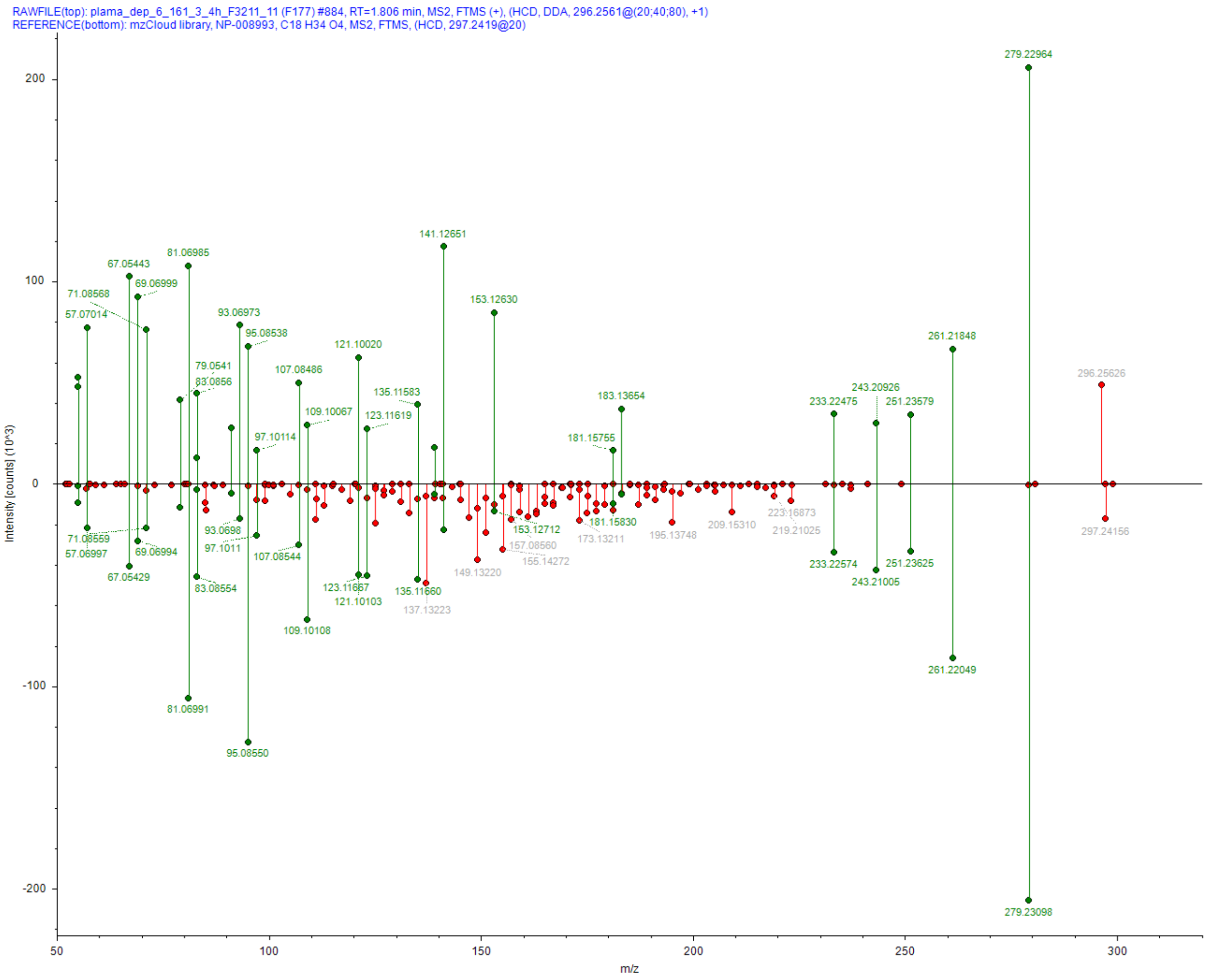

Figure S5. Comparison of the experimental fragmentation spectrum of (2E,4E)-N-(2-Hydroxy-2-methylpropyl)-2,4-tetradecadienamide (top) with the spectrum of NP-008993 from the mzCloud database (bottom).
